# Asymptomatic Familial Multiple Cerebral Cavernous Malformation in a 73-Year-Old Woman

**DOI:** 10.1155/2021/9974776

**Published:** 2021-05-21

**Authors:** Klenam Dzefi-Tettey, Emmanuel Kobina Mesi Edzie, Philip Narteh Gorleku, Henry Kusodzi, Abdul Raman Asemah

**Affiliations:** ^1^Department of Radiology, Korle Bu Teaching Hospital, 1 Guggisberg Avenue, Accra, Ghana; ^2^Department of Medical Imaging, School of Medical Sciences, College of Health and Allied Sciences, University of Cape Coast, Cape Coast, Ghana

## Abstract

Cerebral cavernous malformations (CCMs) are dilated blood vessels which can develop sporadically or in familial form and are the commonest malformations of blood vessels in the spinal cord and brain. The familial form is an autosomal dominant gene mutation disorder. This condition can be diagnosed with magnetic resonance imaging (MRI) and computed tomography (CT) scan, but the modality of choice is MRI because of its high sensitivity. We report a case of a 73-year-old woman with an asymptomatic multiple familial cerebral cavernous malformation (FCCM) which was previously misdiagnosed as multiple cerebral metastases on CT scan. A brain MRI performed correctly diagnosed her condition as FCCM based on the typical MRI appearances. In order not to misdiagnose brain lesions like CCM on CT scan, for cerebral metastases in resource-poor settings, radiologists must recommend advanced imaging modalities like MRI for further evaluation, thereby avoiding unnecessary invasive surgical biopsies.

## 1. Introduction

Cerebral cavernous malformations (CCMs) are the most common malformations of blood vessels and can be seen in various parts of the brain and the spinal cord. CCMs are clusters of abnormal blood vessels that are enlarged and irregular in structure [1–3]. Many patients with CCMs are asymptomatic; hence, its real prevalence is unknown [4]. However, based on autopsy studies, the incidence of CCMs is reported to range from 0.4% to 0.8% in the general population [5–7]. The advancement in the central nervous system (CNS) imaging has led to the incidental diagnosis of CCMs with the use of modalities such as computed tomography (CT) scan and magnetic resonance imaging (MRI), with the latter being the more effective imaging modality for detecting CCMs, especially the T2 Gradient Recalled Echo (GRE)/T2∗ and Susceptibility-Weighted Imaging (SWI) sequences.

CCMs are classified into two forms, namely, sporadic and familial forms. The sporadic form is often associated with a single isolated lesion whilst the familial form is associated with multiple lesions and mutations in three genes, namely, K-Rev Interaction Trapped 1 (KRIT1)/CCM1, CCM2, and Programmed Cell Death 10 (PCD10) also known as CCM3. Approximately 40-60% of familial cerebral cavernous malformations (FCCMs) are inherited in an autosomal dominant pattern due to a heterozygous mutation in one of the three genes [8, 9].

Although CCM is often asymptomatic, people with this condition may present with clinical complaints such as seizures, headaches, deficits in neurological function, and cerebral hemorrhage [10]. The incidence of asymptomatic CCMs ranges between 9% and 88% in patients presenting with no history of hemorrhage. In familial CCMs, 6.5% and 13% per patient-year have been reported as symptomatic and asymptomatic hemorrhage rates, respectively, with 1.1% per lesion per year, causing FCCM to occur in multiples [1, 11]. The multiple nature of this condition can be misdiagnosed as brain metastasis if the appropriate imaging modality is not used and the radiologist interpreting the images is not experienced, hence this case report.

## 2. Case Report

A 73-year-old woman was referred to the MRI unit of the department of radiology, Korle Bu Teaching Hospital, for brain MRI on account of suspected cerebral metastases. Prior to this, in 2019, she had an invasive moderately differentiated adenocarcinoma of the sigmoid colon and had sigmoid colon resection and end-to-end colorectal anastomosis. She underwent chemotherapy and was doing well, but yet to receive radiotherapy. She was not a known hypertensive and diabetic and had no history of amyloidosis. She is the only child of her deceased parents; the cause of death of her father was cerebrovascular accident. She has one child.

**Figure 1:**
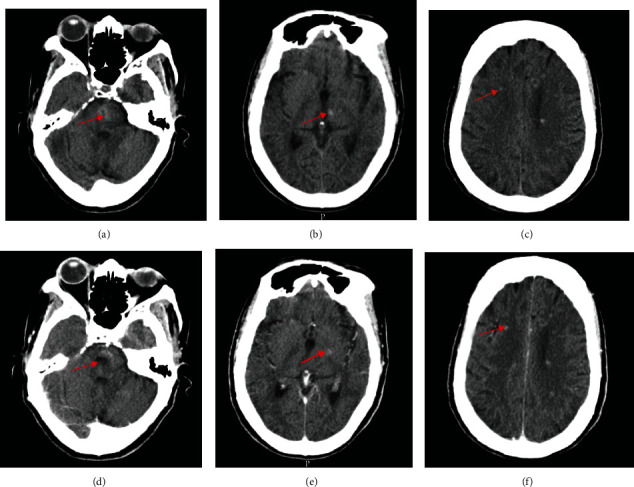
Axial (a–c) unenhanced and (d–f) enhanced cerebral computed tomography images showing multiple, diffuse hyperdense lesions in the infratentorial (pons), basal ganglia, and other supratentorial regions of the brain (red arrows) suggesting multiple cerebral cavernomas.

**Figure 2:**
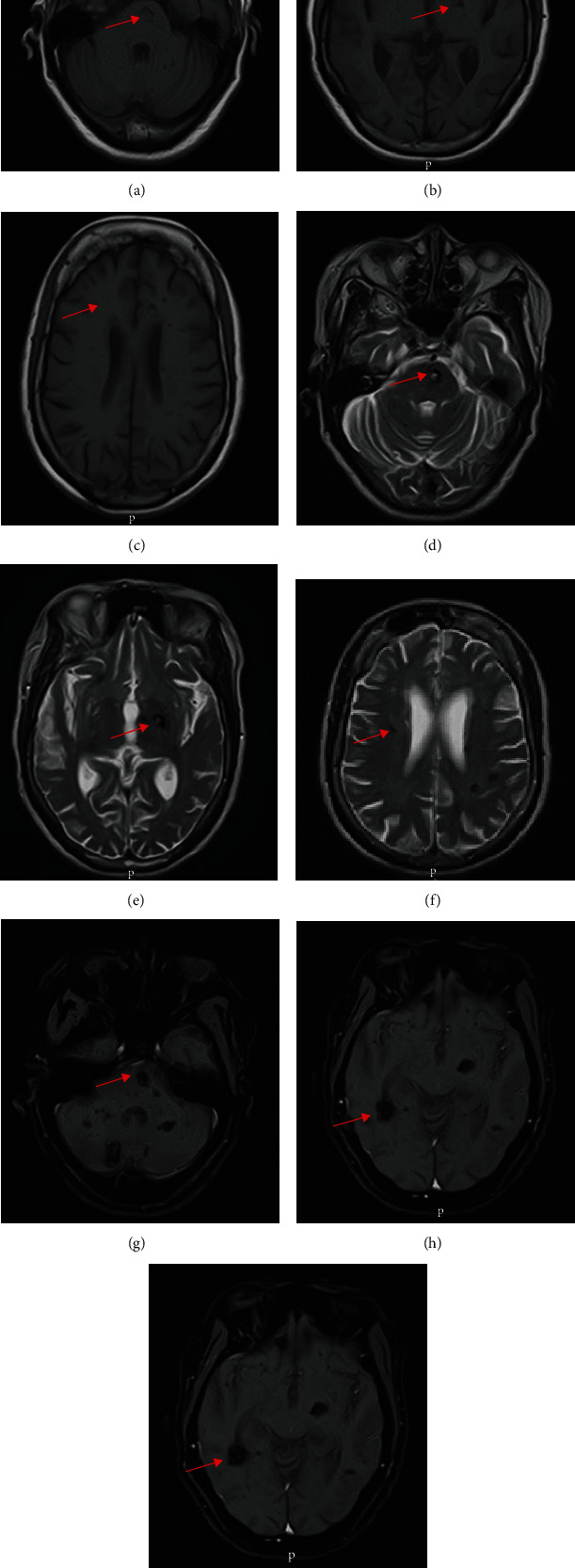
Axial brain MRI: (a–c) T1W, (d–f) T2W, and (g–i) T1W postgadolinium enhanced images showing multiple diffuse cerebellar, pons, bilateral basal ganglia, and cerebral lesions of varying sizes (red arrows); the larger lesions on T1W and T2W images showed hyperintense core and peripheral rim of hypointensity due to hemosiderin in keeping with multiple cerebral cavernomas. No enhancement was noted postgadolinium contrast administration (g–i).

**Figure 3:**
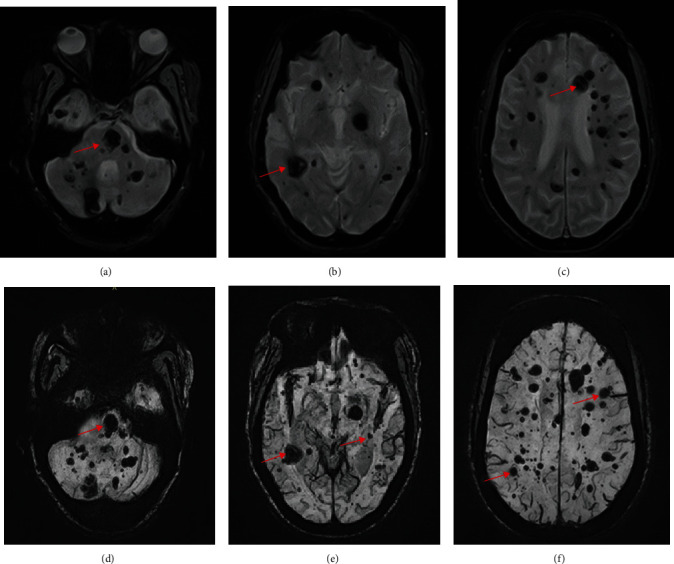
Axial brain MRI: (a–c) GRE/T2∗ and (d–f) SWI showing multiple diffuse cerebellar, pons, midbrain, bilateral basal ganglia, and cerebral lesions of varying sizes (red arrows) which became more prominent due to susceptibility artefacts (blooming) in keeping with multiple cerebral cavernomas.

As part of her metastatic workup, a brain CT scan was done at an outside hospital. The findings (Figure 1) were misdiagnosed as cerebral metastases. She was later referred to do a brain MRI in our facility. We reviewed the CT scan images and found diffuse hyperdense lesions in the infratentorial (pons), basal ganglia, and other supratentorial regions of the brain, which did not have the classic features of cerebral metastases like perilesional edema and ring enhancement. The MRI of the brain performed with T1- and T2-weighted imaging (T1WI and T2WI), Fluid Attenuation Inversion Recovery (FLAIR), GRE/T2∗, and Susceptibility-Weighted Imaging (SWI) sequences revealed multiple diffuse bilateral cerebral, basal ganglia, midbrain, pons, and cerebellar lesions of varying sizes ranging from 2 mm to 20 mm. No enhancement was noted postgadolinium contrast administration. No other associated abnormalities like arteriovenous malformations, aneurysms, or capillary telangiectasia were visualized (Figures 2 and 3).

The typical MRI appearance and the high number of the lesions (approximately more than 70) were in keeping with multiple FCCMs. She was counseled on the associated chances of having spinal cord cavernomas, genetic inheritance of the condition, and the risk of cerebral hemorrhage among others. She therefore consented for MRI of the spine which did not reveal any associated cavernomas of the spinal cord. Her only son also underwent MRI of the brain and spinal cord, which were unremarkable. The genetic testing could not be done to determine mutation in any of the genes associated with FCCMs due to unavailability of the testing facility in the country.

## 3. Discussion

CCMs are the most common type of vascular lesions in the central nervous system, comprising 5%–15% of all vascular malformations. Prior to the use of MRI in diagnostic imaging, symptomatic lesions were more dominant among the 163 CCM cases reported in literature as at 1976, and with the availability of MRI, at least 40% of CCMs are identified incidentally [11, 12]. Out of an incidence rate of 0.4% for cavernomas, 18.7%-20% of all cases are estimated to be multiple cerebral cavernomas [5, 7].

FCCM syndrome is defined as the presence of multiple CCM (typically five or more) or when at least two family members have CCM or a mutation in one of the three genes causing FCCM [10]. Our 73-year-old patient met one of these criteria by having multiple cerebral cavernomas which were approximately more than 70. CCMs are dynamic in nature; hence, new lesions may appear at a rate between 0.2 and 0.4 lesions per patient per year. This explains why our patient who was quite old presented with diffuse CCMs [10]. FCCM accounts for only a small fraction of CCMs; thus, they are uncommon yet underdiagnosed or misdiagnosed as in this case [10]. Since the diagnosis of CCMs can be difficult, new MRI sequences such as GRE/T2∗ and SWI have been developed and being used in addition to the standard T1W, T2W, and FLAIR sequences, whilst diffusion tensor imaging and functional MRI are being applied to the preoperative and intraoperative management of these lesions [5, 13, 14].

In our patient, the larger lesions on T1W, T2W, and FLAIR showed hyperintense core and peripheral rim of hypointensity due to hemosiderin, and this suggests that there was a recent minimal bleeding but she remained asymptomatic. The lesions became more prominent on GRE/T2∗ and SWI due to signal loss and blooming artefacts, and these two sequences are crucial in detecting the cavernomas. MRI can also characterize cavernomas into four types, namely, type 1, 2, 3, and 4 lesions, as originally reported by Zabramski et al. [15]. Type 1 lesions show hyperintense core on spin echo T1- and T2-weighted images, and this suggests recent hemorrhage as seen in our patient. A type 2 lesion appear on T1 and T2W imaging as reticulated mixed signal core and has surrounding hypointense rim. Type 3 lesions are hypointense on T1W imaging, hypointense with hypointense rim on T2W imaging, and blooming on GRE/T2∗ sequence whilst type 4 lesions appear as punctate hypointense lesions on GRE/T2∗ or SWI and could not be visualized on T1- and T2-weighted images, and this was the case in our patient as most of the lesions could not be visualized on her TI- and T2-weighted images and are very conspicuous on the GRE/T2∗ and SWI [5, 15].

From the descriptions of the types of lesions, it is obvious that our patient has all the 4 types. Some authors also described type 4 lesions as small malformations that can only be seen on GRE/T2∗ as hypointense foci and are thought to be capillary telangiectasias [12]. It is also documented that location of the lesions in the brain either supratentorial (75%) or infratentorial (25%) is of great clinical importance and almost half of the infratentorial lesions occur in the brainstem, and these are frequently associated with symptoms related to hemorrhage, whilst supratentorial lesions are frequently associated with seizures [10].

It is also important to differentiate multiple cerebral cavernomas from amyloid angiopathy. On imaging, these have typical subcortical distribution and our patient's lesions do not show this type of distribution. Hypertensive microbleeds are another differential diagnosis, but the patient was not a known hypertensive and hypertensive bleeds have a predilection for basal ganglia region [10]. Hemorrhagic cerebral metastases are very rare, and there should be a history of a primary malignancy like melanoma. Diffuse axonal injury is a differential diagnosis, but these lesions are distributed usually at the grey–white matter junction and there should be a previous history of severe trauma. Neurocysticercosis lesions are typically smaller and uniform, and in addition, they may become calcified with time. These calcifications can be confirmed on brain CT scan, GRE/T2∗, and SWI. Radiation-induced cavernous malformations also mimic FCCMs [10].

Generally, 50% of patients with FCCMs remain asymptomatic throughout their entire lives as seen in our patient partly due to the slow course of FCCMs. Conservative management and symptomatic treatment are highly recommended for this condition. A surgical intervention is only recommended in patients with multiple lesions, hemorrhage, progressive neurological deficits, and seizures [9]. Our patient was asymptomatic; hence, no management was recommended. Patients with FCCM should be carefully followed up even if they are asymptomatic.

## 4. Conclusion

In order not to misdiagnose brain lesions like cerebral cavernous malformations on CT scan for cerebral metastases, radiologists must recommend advanced imaging modalities like MRI for further evaluation.

MRI is more sensitive in the detection of CCMs than brain CT scan. Hence, radiologists can use the typical MRI features to diagnose CCMs, thereby avoiding unnecessary invasive surgical biopsies and be aware of the mimics of multiple CCMs.

## Data Availability

Data employed to back this study may be obtained upon a formal application to the chairperson of the medical research division of the radiology department of the KBTH: P.O. Box KB77, Korle Bu, Accra, Ghana; e-mail: rdo@kbth.gov.gh.
